# Measuring inconsistency in research ethics committee review

**DOI:** 10.1186/s12910-017-0224-7

**Published:** 2017-11-28

**Authors:** Samantha Trace, Simon Erik Kolstoe

**Affiliations:** Institute of Biomedical and Biomolecular Science, University of Portsmouth, King Henry Building, Portsmouth, PO1 2DY UK

**Keywords:** Research ethics committees, Consistency, Quality, Variation, Research

## Abstract

**Background:**

The review of human participant research by Research Ethics Committees (RECs) or Institutional Review Boards (IRBs) is a complex multi-faceted process that cannot be reduced to an algorithm. However, this does not give RECs/ IRBs permission to be inconsistent in their specific requirements to researchers or in their final opinions. In England the Health Research Authority (HRA) coordinates 67 committees, and has adopted a consistency improvement plan including a process called “Shared Ethical Debate” (ShED) where multiple committees review the same project. Committee reviews are compared for consistency by analysing the resulting minutes.

**Methods:**

We present a description of the ShED process. We report an analysis of minutes created by research ethics committees participating in two ShED exercises, and compare them to minutes produced in a published “mystery shopper” exercise. We propose a consistency score by defining top themes for each exercise, and calculating the ratio between top themes and total themes identified by each committee for each ShED exercise.

**Results:**

Our analysis highlights qualitative differences between the ShED 19, ShED 20 and “mystery shopper” exercises. The quantitative measure of consistency showed only one committee across the three exercises with more than half its total themes as top themes (ratio of 0.6). The average consistency scores for the three exercises were 0.23 (ShED19), 0.35 (ShED20) and 0.32 (mystery shopper). There is a statistically significant difference between the ShED 19 exercise, and the ShED 20 and mystery shopper exercises.

**Conclusions:**

ShED exercises are effective in identifying inconsistency between ethics committees and we describe a scoring method that could be used to quantify this. However, whilst a level of inconsistency is probably inevitable in research ethics committee reviews, studies must move beyond the ShED methodology to understand why inconsistency occurs, and what an acceptable level of inconsistency might be.

**Electronic supplementary material:**

The online version of this article (10.1186/s12910-017-0224-7) contains supplementary material, which is available to authorized users.

## Background

Research ethics committees (RECs) have two main aims: 1) to protect the rights, safety, dignity and well-being of research participants; and 2) to facilitate and promote ethical research that is of potential benefit to participants, science and society. [1] However, a number of empirical studies have shown that Research Ethics Committees (and Institutional Review Boards (IRBs)) are inconsistent in how they go about achieving these aims [2–8]. Along with being ethically concerning (by potentially leading to the unequal treatment of participants in studies) inconsistency is problematic in larger administrations with multiple ethics committees because researchers are quick to complain if they receive inconsistent treatment or perceive their projects being delayed for arbitrary reasons [9]. Administrative bodies have drawn upon previous experience in ethics review to produce standard operating procedures, checklists and guidance to help committees identify and deal consistently with common issues [10]. The general success of such guidance has led some to suggest that the proper role for a REC is to ensure administrative “code-consistency” [11], and that broader or more abstract ethical issues may be too difficult to be addressed by RECs. Whilst we agree that code consistency is certainly easier to address, we believe that ethics committees necessarily discover and grapple with more abstract ethical problems as they review new research that pushes the boundaries of knowledge. Whilst a certain level of inconsistency might be expected within any process relying upon discourse ethics, the challenge is to measure inconsistency, understand why it occurs, and then determine what level is acceptable.

The main practical task of a research ethics committee is to review written experimental protocols, often in the form of ethics application forms. After deliberation within the committee a decision is provided, and although the language used in different systems can vary, decisions are normally in the form of a direct “favourable”, “unfavourable”, or more commonly a “provisional” opinion accompanied by a list of concerns detailing changes required before a final ethics opinion can be granted. A number of previous studies have shown that ethics committees are relatively consistent in the final opinions they give, but are often inconsistent in the reasons given for their opinion [2, 3, 5, 6]. In cases of provisional opinions this is an annoyance to the researchers who may experience similar protocols being altered in seemingly arbitrary ways. In cases of unfavourable opinions this is ethically problematic as it suggests committees often do not have clear reasons why they are concerned about projects, perhaps acting on intuition [6] rather than a common set of ethical principles. This issue is also a concern to administrative authorities overseeing ethics committees who feel a practical obligation to ensure that decisions are as consistent as possible across their many committees.

In England 67 RECs are overseen by the Health Research Authority (HRA) who provide an administrative structure for research ethics review of projects conducted in the National Health Service, in adult social care, or subject to certain legislation. The governance [12] and practical arrangements [13] for HRA RECs are described in detail and freely available online. Briefly, committees consist of up to eighteen members including both expert (professionally involved in medicine or medical research) and lay members. Committees meet once a month and consider approximately 6 studies in a four-hour meeting. Research teams are invited to answer questions from the ethics committee, and minutes summarising the discussion and listing the points that need addressing by the researchers are taken by a HRA “REC manager” who composes letters to the researchers based on these minutes, and handles further correspondence in liaison with the REC chair. A “proportionate review” process also exists for less contentious studies, but this is not the focus of this paper. In order to promote consistency, the HRA has developed review and minute taking templates to try and direct committees’ focus towards the main ethical issues that might arise in any given project. This involved the refinement of ten ethical “review domains” that a committee needs to consider when reviewing a study (Table 1) [14]. The process has been particularly important to promote consistency in the case of studies that fall under certain UK or European legislation that have statutory requirements for review (e.g. EU clinical trial regulations or the UK Human Tissue Act amongst others).

**Table 1:** Review domains specified by the HRA

1. Social or scientific value; scientific design and conduct of the study (including involvement of patients, service users and the public, in the design, management, and undertaking of the research).
2. Recruitment arrangements and access to health information, and fair research participant selection
3. Favourable risk benefit ratio; anticipated benefits/risks for research participants (present and future)
4. Care and protection of research participants; respect for potential and enrolled research participants’ welfare & dignity
5. Informed consent process and the adequacy and completeness of research participant information
6. Suitability of the applicant and supporting staff
7. Independent review
8. Suitability of supporting information
9. Other general comments
10. Consider and confirm the suitability of the summary of the study

Pre-dating the review templates, the HRA has also run shared ethical debate (ShED) exercises a few times each year. This process originally started as a single-issue ethical debate where committees were asked to consider a specific ethical question, but has since evolved into a more complex process. The most recent ShEDs have consisted of gaining permission from researchers to circulate real applications (that have already been through the system and gained a final opinion) to around twenty committees. Participating RECs are encouraged to review the study at their meeting and then send a copy of their minutes containing their decision to the ShED coordinator to perform a basic content and/or thematic analysis. This allows a convenient comparison between committees. Results of the exercise are circulated to the participating committees to help inform them of their performance, but also considered more widely within the HRA to set future training and policy agendas. The HRA make it clear that they view this process as an internal audit of their systems stating:

*“The ShED process encourages ethical debate across committees and is used to look for trends in decision-making and key ethical themes, which can then be used to review and improve consistency through guidance and training.”* [15].

**Fig. 1:**
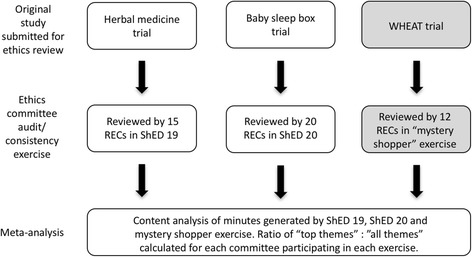
Overview of methods highlighting the initial studies, the ethics committee exercises and our analysis. Analysis in grey boxes performed by Gale et al.

In this paper, we present an overview and analysis of two ShED exercises 19 and 20, and then compare them to a “mystery shopper” exercise also performed using NHS ethics committees. This latter exercise involved a research team presenting their study (detailed below) to twelve ethics committees using a “mystery shopper” methodology similar to that used by the market research community. After drawing out some observations from these three exercises we propose a numerical method for quantifying inconsistency. A graphical overview of the work described is displayed in Fig. 1. We note the differences between measuring inconsistency, explaining why it occurs, and determining what level of inconsistency might be expected between RECs.

## Methods

### Identifying themes

In early 2016 we were asked by the HRA to analyse the results of the ShED 19 and 20 exercises. We were provided with the documentation for the study being reviewed as the focus of both ShEDs (application form, participant information, consent forms etc.) along with the minutes produced by each committee participating in the ShED. It is of note that minutes produced by HRA committees contain a summary of the committee deliberations as well as the points that require addressing by the researcher. There was no further communication between the RECs and the researchers presenting the ShED studies, so no subsequent correspondence was available for analysis. Soon after producing our reports a “mystery shopper” study was published by Gale et al. [16] describing their interaction with twelve HRA administered committees reviewing a randomised control trial in preterm infants (called WHEAT – “With Holding Enteral feeds Around packed red cell Transfusion”). We were also able to analyse the raw data from this study in the same way as we did for the ShED exercises as full ethics committee minutes and correspondence were included in the supplementary data to the published paper. Using NVivo [17], a content analysis was conducted on each set of minutes for each of the three studies (using a grounded theory approach - further details provided in the Additional file 1). For this piece of work we did not distinguish between positive, neutral and negative comments, as we were interested solely in which themes committees discussed.

### Scoring

Following the content analysis, we defined any theme discussed by more than half the committees as a “top theme” and calculated the ratio between top themes and all themes per committee for each of the three exercises. Using this ratio, the top score for a committee that only identified top themes would be one, and a committee that identified no top themes would be zero. By calculating a ratio this metric corrects for the total number of themes identified by committees. Quantitative analysis was performed using MS Excel and IBM SPSS v24.0.

## Results

In the following sections we present brief observations from each of the three individual exercises (ShED 19, ShED 20 & WHEAT) before describing the scoring results.

### Overview of ShED 19 and themes identified by committees participating in this exercise

Fifteen HRA RECs participated in ShED 19, a study involving the use of Chinese herbal medicine (CHM) for controlling recurrent urinary tract infections. Although the researchers designing the project were well qualified in conventional medicine, and had tried to design a conventional placebo controlled, double-blinded and randomised study, the use of alternative medicine still raised concern amongst committees. The content analysis of the 15 sets of minutes identified 315 individual comments that could be grouped into 130 themes, that were then grouped into one of the 10 ethical domains (described in Table 1). The eight top themes identified by more than half the committees are listed in Table 2. Most notable were concerns about constructing the objectives/arms of the trial and then randomising between those who would be receiving a talking therapy approach from the herbal medicine practitioners, and those who would attend conventional, shorter, GP appointments. The researchers were intending to include an arm trying to control for the type of clinical session, but the additional arm raised concerns from some committees who said that the study was trying to examine too many things. Indeed, a point alluded to by many committees was whether a placebo control is ever relevant when conducting research on alternative medicine. Similarly, of the top eight themes, three were related to the differences in accepted practice between alternative and conventional medicine, suggesting that discussions had considered wider cultural factors rather than just the protocol itself.

**Table 2:** Top themes defined as those identified by more than half the committees in the ShED19, ShED20 and WHEAT exercises

Theme	Number of RECs	% of RECs
*ShED 19*		
Query or statement regarding overall objectives & outcomes	14	93%
Comment over worsening of UTI and/or detecting side effects	10	67%
PIS: Explanation of placebo and/or randomisation	10	67%
Clarity over insurance & indemnity information	9	60%
Methodological comment over controls, placebo &/or randomisation	8	53%
Methodological comment over design of arms in trial	8	53%
Query regarding collection of human tissue	8	53%
PIS: Further advice if symptoms persist	8	53%
*ShED 20*		
Comments on use of Safer Sleep Box	19	95%
Limits of confidentiality being clear to participants	16	80%
Inclusion criteria of trial	15	75%
Inclusion of vouchers as incentive	13	65%
Data protection concerns	13	65%
PIS: Typos, grammar, formatting and re-titling	12	60%
Recruitment method	12	60%
Positioning of box during use	11	55%
Comments on size of participant group	11	55%
Independent Review	11	55%
Comments on questionnaires	10	50%
Training and information on safer sleep box for participants	10	50%
Methodology and basic nature of study	10	50%
Scientific justification of the study	10	50%
*WHEAT*		
Opt Out Consent: any mention	12	100%
PIS: comments on potentially coercive wording	9	92%
Opt Out Consent: Comments on the ethics of the basic principle	8	67%
PIS: Suitable description of risks and benefits	7	58%
Opt-Out Consent: how this is to be recorded	7	58%
Opt-Out Consent: Hard copy for patients/clinicians	7	58%
Patient Public Involvement	6	50%

A complete list of themes is contained in the Additional file 1

The second main group of top themes seemed to be concerns regarding the safety of patients in terms of both urinary tract infection progression and side effects/purity of the herbal medicine. These were both issues that the researchers had tried to address in their documentation, although two thirds of committees still raised concerns, and thus would perhaps have benefitted from being reassured by speaking directly with the researchers. Unlike a normal review this ShED exercise did not allow committees to raise and potentially resolve concerns directly with the researchers. Indeed, the research team had gone to significant lengths to source a consistent and regulated supply of the herbs, but a number of committees were not satisfied, with five wanting evidence of MHRA approval. Another top theme identified by committees was whether “relevant material” (as defined by the UK Human Tissue Act) was being collected. This was an interesting issue as it highlighted a difference in interpretation of the term “storage” within the act.

**Fig. 2:**
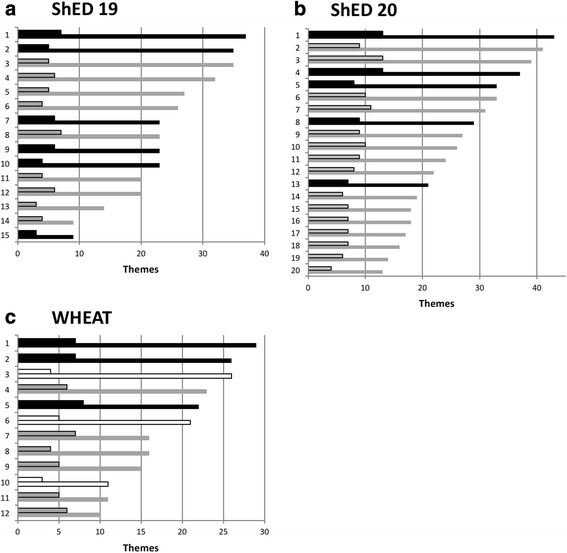
Number of themes and top themes identified by each committee for ShEDs 19 (**a**) and 20 (**b**), and the WHEAT trial (**c**). Black denotes unfavourable opinion, grey provisional and white favourable. Smaller outlined bars the top themes. Numbers on y axis denote committee identifier and not the same committees between studies

The majority of the other themes (i.e. non-top themes) were mainly administrative in nature revolving around either updates and/or clarifications to the participant information sheets (37 themes), consent forms (11 themes) or various other aspects of the documentation. Interestingly only four of the themes relating to the participant information sheet, and seven other themes, were picked up by more than a third of the committees, indicating that there may have been an element of committee or even individual member preference regarding many of these issues. Overall, as only one out of the 130 themes were picked up by all but one committee, eight by more than half, and 24 by more than a third, it seems that the majority of the total themes were unlikely to be considered significant by the majority of RECs. Of the 15 committees that participated in this study nine gave provisional opinions and six unfavourable opinions (see Fig. 2). It was unclear from the specific minutes precisely why some committees chose to give an unfavourable over a provisional decision.

### Overview of ShED 20 and themes identified by committees participating in this exercise

ShED20 was a topical study looking at the feasibility of “safer sleep boxes” for use with new-born infants co-sleeping with their parent(s). The 20 committees participating in this ShED produced 530 comments that were reduced to 87 themes and 14 top themes (Table 2). Although there were almost twice as many top themes as in ShED19, there were far fewer themes overall. As with ShED19 the committees did not have the opportunity to interview the researchers.

The majority of concerns related to the use of the safer sleep box. Whilst a few committees did comment that this system was used in other countries and thus was worthwhile to trial in the UK, the lack of familiarity with the box may have influenced the number of concerns. Perhaps unsurprisingly the inclusion of vulnerable young mothers and children did feature in many committee discussions, with some committees viewing this as a problem, but others viewing it as a necessary part of the research.

Whilst all the minutes were written using the HRA minutes template, the content was seldom organised under the most appropriate headings. Indeed, it was not uncommon for phrases such as “No issues were raised in this area” to appear under a specific heading, but then issues related to the area appearing in the reasons for decision section. This may have been due to the way discussions were chaired, perhaps focusing on concerns as they arose in the discussion rather than structuring the discussion around the minutes’ template. Likewise, some concerns touched on a number of different areas and themes making it difficult to categorise effectively. Indeed, in the majority of minutes the decision section was far more developed than the discussion section, perhaps reflecting the emphasis of providing clear feedback to researchers rather than accurately categorising all aspects of the discussion.

Of the 87 themes identified only comments regarding the safe use of the sleep box were made by all but one committee. 16 of the 20 committees had concerns about confidentiality and more general data protection, 15 about the inclusion criteria, and 13 about the use of vouchers as payment. A further nine themes were commented on by at least half the committees, meaning that the other 73 themes were commented on by fewer than half the committees. 15 of the 20 committees participating in this study gave a provisional opinion and five an unfavourable. The reasons for the unfavourable opinions were either on safety grounds or the inclusion of vulnerable mothers.

### Overview of the “mystery shopper” WHEAT trial

During our analysis of the two ShED studies a “mystery shopper” exercise using 12 HRA committees was published in the literature as an opportunistic addition to a larger paediatric project examining *“a comparative- effectiveness, randomised controlled trial comparing two widely used blood transfusion practices in preterm infants*” [16]. The research team who published this study were trying to understand how ethics committees would view four specific aspects of their trial design:

(i) *point-of-care design using electronic patient records for patient identification, randomisation and data acquisition,*
(ii) *short two-page information sheet;*
(iii) *explicit mention of possible inclusion benefit;*
(iv) *opt-out consent with enrolment as the default.*

Similar to the ShED exercises, this single study was presented to twelve ethics committees and the resulting minutes were analysed by the WHEAT team in light of the above four aspects. The study differed from the ShED exercises in that the committees were blinded to this being an ethical investigation, and a researcher was able to attend six of the committee meetings in person (and was available to speak over the phone to the other six - although only four took advantage of this). The research team was also prepared to adapt their study in light of the committee feedback except for the four specific ethical issues where they intentionally defended their position. The methodology used in this study was therefore far closer to a real review than the ShED exercises, and hence the term “mystery shopper”. One committee gave a favourable opinion outright, one an unfavourable without requesting a response, whilst the other ten required written responses. Of these, eight subsequently gave a favourable opinion and two unfavourable (Fig. 2). No committees queried the point-of-care design, and one queried the shorter information sheet although subsequently accepted an argument for the benefit of shorter information sheets. Nine committees raised concerns about including a statement in the participant information regarding the benefits of participating in research more generally, although eight subsequently accepted a different form of wording for this. However, the three committees that gave an unfavourable opinion all considered the opt-out consent ethically invalid, two indicating that they considered opt-out consent to be inappropriate for any randomised trial. The authors formed a number of helpful conclusions specific to trial design in their particular population, and whilst being generally praising of the HRA’s support for research ethics committees and their attempts to promote consistency, commented that inconsistency is still commonplace and that this:

*“serves neither patients nor researchers well and risks breakdowns in trust. This highlights the importance of sound policies to improve REC consistency.”*

Following publication of this work we downloaded all the sets of minutes from the supplementary data of the original paper, and then performed a content analysis identical to that used in our analysis of the ShED exercises. The resulting themes are listed in Table 2 and a complete list can be found in the Additional file 1 to this paper.

### Scoring committee reviews

**Fig. 3:**
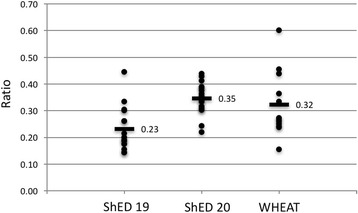
Distribution of the calculated ratios (y axis) for the three exercises with mean values labelled and shown as bars

Across the three exercises (ShED19, ShED20 & WHEAT) the number of themes raised by committees differed by between three and four-fold. Analysis of the number of themes against outcome opinions (favourable, unfavourable etc.) was not possible due to the lack of unfavourable outcomes. Committees giving an unfavourable opinion did seem to comment on more themes, but not enough committees were included to analyse this statistically. In thinking about how to analyse our results further it occurred to us that it may be possible to create a numerical measure of inconsistency as, despite the large number of ethical themes identified by RECs, only a few themes were identified by more than half of the committees participating in each exercise. We therefore defined any theme discussed by more than half the committees as a “top theme” and calculated the ratio between top themes and all themes per committee for each of the three exercises. Using this ratio, the top score for a committee that only identified top themes would be one, and a committee that identified no top themes would be zero. Calculating this for all committees involved in the three exercises provided the ranking shown in Table 3. Only one committee across the three exercises had more than half its total themes as top themes (WHEAT committee 12) with a ratio of 0.6. The lowest score was 0.14 (ShED19 committees 2 and 3) indicating that only 14% of issues raised by these two committees were issues raised by more than half the other committees reviewing the identical project. By calculating a ratio this metric corrected for the total number of themes identified by committees. The ratios for each exercise were normally distributed according to the Shapiro-Wilk test (all have *p* > 0.05). Committees reviewing ShED19 had lower ratios (mean of 0.23, s.d. 0.08) than ShED20 (mean of 0.35, s.d. 0.06) and WHEAT (mean of 0.32, s.d. 0.12) (see Fig. 3). Tukey’s post hoc test revealed that ShED 19 was on average significantly different from both ShED 20 and WHEAT with *p*-values of 0.001 and 0.025 respectively. ShED 20 was, however, not on average significantly different from WHEAT [*p* = 0.768].

**Table 3:** Calculated ratios of top themes to all themes for committees participating in the three exercises

Exercise	Committee identifier	Top themes	All themes	Top theme: All themes
WHEAT	12	6	10	0.60
WHEAT	11	5	11	0.45
ShED19	14	4	9	0.44
ShED20	18	7	16	0.44
WHEAT	7	7	16	0.44
ShED20	19	6	14	0.43
ShED20	17	7	17	0.41
ShED20	16	7	18	0.39
ShED20	15	7	18	0.39
ShED20	10	10	26	0.38
ShED20	11	9	24	0.38
ShED20	12	8	22	0.36
WHEAT	5	8	22	0.36
ShED20	7	11	31	0.35
ShED20	4	13	37	0.35
ShED19	15	3	9	0.33
ShED20	13	7	21	0.33
ShED20	9	9	27	0.33
ShED20	3	13	39	0.33
WHEAT	9	5	15	0.33
ShED20	14	6	19	0.32
ShED20	8	9	29	0.31
ShED19	8	7	23	0.30
ShED20	6	10	33	0.30
ShED20	1	13	43	0.30
ShED19	12	6	20	0.30
WHEAT	10	3	11	0.27
WHEAT	2	7	26	0.27
ShED19	9	6	23	0.26
ShED19	7	6	23	0.26
WHEAT	4	6	23	0.26
WHEAT	8	4	16	0.25
ShED20	5	8	33	0.24
WHEAT	1	7	29	0.24
WHEAT	6	5	21	0.24
ShED20	2	9	41	0.22
ShED19	13	3	14	0.21
ShED19	11	4	20	0.20
ShED19	1	7	37	0.19
ShED19	4	6	32	0.19
ShED19	5	5	27	0.19
ShED19	10	4	23	0.17
ShED19	6	4	26	0.15
WHEAT	3	4	26	0.15
ShED19	3	5	35	0.14
ShED19	2	5	35	0.14

## Discussion

This work was based on a content analysis of minutes produced by three separate exercises designed to compare research ethics committee consistency. Our results add to the evidence [5, 7, 16, 18–21] that inconsistency does occur in research ethics review. As mentioned, one approach to addressing inconsistency has been to distinguish between code and ethical consistency [11], arguing that the role of those overseeing committees should be to only try and ensure consistent processes and application of codes. This is an attractive and topical proposition (especially to organisations administering RECs) as it seems achievable through refining standard operating procedures and providing additional guidance or training. In this respect, the ShED exercises did identify inconsistency in the application of guidance (for instance application of the UK Human Tissue Act in ShED19). Our scoring metric was able to produce a numerical measurement of such inconsistency and show that committee performance varies widely. Comparison of scores between the studies showed that the ShED19 ratios were significantly different compared to the ShED20 and WHEAT studies. This observation is probably due to the particularly contentious nature of the study under review (Chinese Herbal Medicine), providing a warning that inconsistency is probably also dependent upon the nature and complexity of the studies being used for REC comparison exercises. As a result, if the aim is to assess committee performance, comparing committees between exercises is perhaps not as relevant as comparing committees within an exercise. If a committee is found to be a significant outlier within an exercise, this could suggest the need for further training or mentoring of committee members.

When analysing the scores more closely it was a surprise that only one committee had a top themes: all themes ratio of above 50%. Although anecdotal experience by the authors from chairing and attending RECs suggest that often many ethically insignificant issues are discussed at meetings, the results here provide firm quantitative evidence that committees deliberate extensively on subjects not considered ethically problematic by other, similarly constituted committees. As the minutes being analysed did not include time stamps it is impossible to accurately determine how long discussions took, but given that a typical study is only allotted 45 min in a HRA REC meeting agenda, it is probably safe to conclude that much time is taken up discussing (or noting) less significant ethical issues. Although this conclusion is perhaps to be expected given the (sometimes published [9]) complaints from research teams, it is helpful to put a number on such inconsistency to provide a benchmark against which future interventions could be tested. However, as the minutes did not provide any indication of how discussions evolved, which members participated or to what extent, it could prove difficult to improve a score without a complete understanding of how the score was obtained.

### Limitations

Both the ShED exercises and our proposed metric rely heavily on the quality and accuracy of the minute taking. Whilst the adoption of minute taking templates based around the ten ethical domains seems to have introduced an element of consistency with regards the structure of minutes, the quality or the written minutes themselves seemed to vary quite widely in terms of grammar, sentence construction and ease of reading. For instance, the presence of comments recorded in inappropriate sections of the minute template indicate that some REC managers may have found it more difficult conforming to the minute taking guidance than others. Anecdotal evidence from REC managers suggests that some dislike the templates, preferring to directly transcribe the meeting as it happens rather than interpreting and re-ordering statements in light of the ten ethical domains. Interestingly there was no clear reasons in any of the three exercises why similarly minuted discussions seemed to result in different final opinions. Again, anecdotal experience of REC meetings suggests that in the absence of the opportunity to correspond with researchers, provisional opinions are preferred over favourable opinions as quite often the REC requires more information before confirming its opinion. This observation is supported by a previous study [22] showing that researchers who attended REC meetings were also more likely to receive a provisional instead of an unfavourable opinion, emphasising the importance of dialogue between the REC and the research team. In this respect, the WHEAT study had an advantage over ShED 19 and 20 because the researchers were able to correspond with the committees and address certain issues. When committees were able to correspond with investigators during the WHEAT exercise all provisional opinions were converted to favourable opinions, and three immediate favourable opinions were given. It is therefore likely that the final decisions given in ShEDs 19 and 20 would have been different if the committees had been able to speak or correspond with the researchers. This represents a significant weakness of the current ShED process, suggesting that the analysis of final opinions as a measure of consistency might not be accurate for this type of exercise. However, as normally the majority of a committee discussion occurs prior to the researchers entering a room, the identification of themes and top themes is still likely to be accurate.

Another interesting observation coming specifically out of the ShED process was the variability in the total themes raised by each committee. Here the knowledge that the committee was participating in a ShED exercise seemed to result in over compensation by some committees, and a curious lack of comments by others. Although an instruction was given by the HRA that the ShED study was to be discussed at the beginning of each REC meeting, there may still have been time pressures to complete the ShED discussion and move onto the real applications. Again, the mystery shopper nature of the WHEAT exercise tried to correct for this.

## Conclusion

The three exercises described here along with our scoring tool were effective in establishing and quantifying the presence of inconsistency between research ethics committees when reviewing identical studies. However, the current ShED process is over reliant upon the written minutes, the exercise is not blinded, and no insight is given as to how discussions develop in the committee room. The “mystery shopper” nature of the WHEAT exercise corrected for some of these methodological issues, but still did not provide an insight into what actually occurs during committee deliberations. Whilst a level of inconsistency is probably to be expected during ethics discussions, simply detecting inconsistency does not explain why it happens, what should be done about it, or even if it is something to be avoided. To explore these latter questions further investigations taking into account social factors and group dynamics need to be developed. This is consistent with previous work in group decision making that has suggested factors such as committee culture, local thinking [23, 24], leadership styles/ability [25], levels of member experience [26] and social interactions [27] play roles in committee decision making. Inconsistency is probably inevitable in research ethics committee review, but to determine how inevitable, studies must now move beyond ShED.

## Additional file

Additional file 1: Qualitative methods and list of themes. Description of analysis methods originally used and tables of all themes identified for each study. (PDF 338 kb)

## Abbreviations

HRA: Health research authority
IRB: Institutional review board
MHRA: Medicines and healthcare products regulatory agency (UK)
REC: Research ethics committee
ShED: Shared ethical debate
