# Supplementary material for: Measuring inconsistency in research ethics committee review
Source: BMC Med Ethics. 2017 Nov 28;18:65. doi: 10.1186/s12910-017-0224-7 (PMC5704399; doi:10.1186/s12910-017-0224-7)
Supplement: Additional file 1: — Qualitative methods and list of themes. Description of analysis methods originally used and tables of all themes identified for each study. (PDF 338 kb) [file 12910_2017_224_MOESM1_ESM.pdf]

Trace, S. & Kolstoe, S.E. (2017) Supplementary Information

1) Qualitative Methods

Minutes were uploaded to the software package NVivo[1] as separate file sources.

Thematic Analysis

A node was created for each of the ten Review Domains outlined in the HRA minute taking guidance:

1. Social or Scientific Value; scientific design and conduct of the study
2. Recruitment Arrangements and access to health information and fair participant selection
3. Favourable Risk Benefit Ratio; anticipated benefits or risks for research participants' welfare and dignity
4. Care and protection of Research participants respect for potential and enrolled research participants' welfare and dignity
5. Informed consent process and the adequacy and completeness of research participant information
6. Suitability of the applicant and supporting staff
7. Independent Review
8. Suitability of supporting information
9. Other general comments
10. Consider and confirm the suitability of the summary of the study

Each set of minutes was then reviewed and every comment (reference) assigned (coded) to one of the ten domain nodes. Headings and template text were not included. References were coded into multiple domain nodes if required (cross-coding).

Content Analysis

Once the initial thematic analysis had been performed each of the domain nodes was inspected individually. New sub-nodes corresponding to themes were then created inside each of the ten domain nodes corresponding to the ideas contained in each reference. For instance, if a reference in domain 1 referred to the use of placebo, a new sub-node (theme) called "Use of Placebo" was created within domain node 1, and any further references from the same or other committees regarding the use of placebo added to this sub-node. As domain node 5 contained a large number of references regarding the Participant Information Sheet (PIS) and consent form, it was helpful to prefix many sub-nodes in this particular domain with either "PIS" or "CONSENT". If, as coding progressed, overlap was found between sub-nodes, similar sub-nodes were merged if required. Similarly, it was occasionally necessary to split sub-nodes if the references previously coded together became too divergent.

Validation

Once coding had been completed the original minutes were checked to ensure that all text had been coded into a domain node and theme sub-node.

[1] NVivo qualitative data analysis Software; QSR International Pty Ltd. Version 10, 2012

2) Complete Themes tables with top themes indicated

ShED 19

Theme	No. of RECs	% of RECs
Query or statement regarding overall objectives & outcomes	14	93%
Comments over worsening of UTI and/or detecting side effects	10	67%
PIS: Explanation of placebo and/or randomisation	10	67%
Clarity over insurance & indemnity information	9	60%
Methodological comments over controls, placebo &/or randomisation	8	53%
Methodological comments over design of arms in study	8	53%
Query regarding collection of human tissue	8	53%
PIS: Further advice if symptoms persist	8	53%
Power & Stats & Analysis	7	47%
CTIMP or MHRA query	7	47%
Contentious nature of CHM	7	47%
PIS: Spelling/grammar corrections	7	47%
Details required of independent review	7	47%
Standardisation of recruitment procedure	6	40%
Safety of CHM	6	40%
Risk benefit ratio	6	40%
Missing CV	6	40%
Application form errors	6	40%
PPI	5	33%
Formulation of CHM	5	33%
Pregnancy test before consent	5	33%
Liver & Kidney tests	5	33%
PIS: More information required (various sections)	5	33%
Qualifications of CHM practitioners	5	33%
Supply comments	4	27%
Personal nature of CHM prescription	4	27%
Issue with GP recruitment letter	4	27%
CONSENT: Audio recording	4	27%
PIS: Information on pregnancy/pregnancy test	4	27%
PIS: Background on CHM	4	27%
PIS: Inadequate, follow HRA advice	4	27%
PIS: More detail on side-effects of CHM	4	27%
Details in information letter to GP	4	27%
Destruction of files	4	27%
Withdrawal Criteria	3	20%
Expand inclusion & exclusion criteria	3	20%
Good inclusion & exclusion criteria	3	20%
Post study availability of CHM	3	20%
CONSENT: Bloods	3	20%
CONSENT: Broad consent clause	3	20%
PIS: Expand statement of what participation involves	3	20%
PIS: Interview details	3	20%
PIS: Contact details	3	20%
PIS: More information on bloods	3	20%

Evidence of GCP training	3	20%
Approval of experienced CI	3	20%
Independent review noted	3	20%
SSA needed for CHM sites	3	20%
Practical comments RE organisation	2	13%
Well justified	2	13%
Trial registration	2	13%
Clarification of recruitment procedures	2	13%
Access to practice lists	2	13%
Issues with confusion of standard care and study procedures	2	13%
Misdiagnosis issues	2	13%
Detection of new UTI's	2	13%
Management of results by GP vs CHM	2	13%
CONSENT: Sample storage	2	13%
CONSENT: Access to records	2	13%
PIS: Withdrawal procedure	2	13%
PIS: Name of ethics committee	2	13%
PIS: More information concerning diary	2	13%
PIS: Not lay friendly	2	13%
PIS: Details of indemnity	2	13%
PIS: Details of future research	2	13%
PIS: Describe difference between CHM and normal treatment	2	13%
PIS: Details of audio recording	2	13%
PIS: Changes to sections/section headings	2	13%
PIS: CHM tea making instructions	2	13%
Nurse as participant	2	13%
Statistical review appropriate	2	13%
Missing questionnaires	2	13%
Lone worker policy	2	13%
Washout period	1	7%
Comments about qualitative elements	1	7%
Confirmed non MCA	1	7%
Long term cost of supplying CHM	1	7%
Funding	1	7%
Request evidence of previous studies	1	7%
Dosage question	1	7%
Blinding & Unblinding	1	7%
Confirm antibiotic resistance is a problem	1	7%
Warning about contraindications	1	7%
Time to consider participation	1	7%
No travel costs	1	7%
Comments about handling of blood results	1	7%
Access to medical notes	1	7%
Specific concern on side effects	1	7%
Safety if new pregnancy	1	7%
Question regarding adverse event handling	1	7%
Withdrawal procedures	1	7%
Suitable confidentiality	1	7%
Post-study check for infection	1	7%

Onerous questionnaire	1	7%
Good management of participants	1	7%
Ethics of placebo	1	7%
Compensation	1	7%
Why all study team needs access to records	1	7%
CONSENT: Unattributed quotes	1	7%
CONSENT: Initial boxes	1	7%
CONSENT: Contacting GP	1	7%
CONSENT: Countersigning form	1	7%
CONSENT: Consent to access of GP notes	1	7%
CONSENT: Specific wording change	1	7%
PIS: General warning RE CHM's.	1	7%
PIS: Details of travel expenses	1	7%
PIS: Concern of PIS being sent in the post	1	7%
PIS: Describe treatment options if not in study	1	7%
PIS: Describe treatment options after study finishes	1	7%
PIS: Medications to avoid	1	7%
PIS: Confirm arrangements for data after trial	1	7%
PIS: Describe how quotations will be use	1	7%
PIS: Make clear GP's not endorsing CHM	1	7%
PIS: State GP's will be informed of participation	1	7%
PIS: Describes taste and side-effects well	1	7%
PIS: State study is a feasibility study	1	7%
PIS: Describe clinics and their locations	1	7%
PIS: Provide contact details of CHM practitioners	1	7%
PIS: Describe details of 6 month follow-up	1	7%
Version numbers issue	1	7%
More information in diary	1	7%
Instructions on medicine in English	1	7%
Blood samples need to be taken by qualified staff	1	7%
Practice nurse compensation	1	7%
Query over faculty of homeopathy award	1	7%
Chief Investigator CV too long	1	7%
Standardisation of consultations between CHM & GP	1	7%
Researcher non-attendance at meeting	1	7%
Contact for participation in future studies	1	7%
Signature issues	1	7%

ShED 20

Theme	No. of RECs	% of RECs
Comments on use of Safer Sleep Box	19	95%
Limits of confidentiality being clear to participants	16	80%
Inclusion criteria of trial	15	75%
Vouchers incentive	13	65%
Data protection concerns general	13	65%
PIS Typos, grammar, formatting and re-titling	12	60%
Recruitment method	12	60%
Positioning of Box during use	11	55%
Comments on large participant group.	11	55%
Independent Review	11	55%
Questionnaires	10	50%
Training and information on Safer Sleep box for participants	10	50%
Methodology and basic nature of study	10	50%
Scientific justification for this study	10	50%
Inclusion of younger mothers	9	45%
Public involvement in the design of the study	9	45%
Sleep diary	9	45%
PIS Limits of confidentiality	9	45%
Safety accreditation of Baby Bed Box	9	45%
Box lids	9	45%
Potential for coercion	9	45%
Assignment of participant and baseline groups not random	9	45%
Involvement of 'At risk' children	8	40%
English speaking as criteria	8	40%
Typographical Errors	8	40%
What happens when things go wrong	8	40%
Concern over mental capacity	8	40%
Inclusion of fathers	8	40%
PIS Ease of understanding	7	35%
Health visitor involvement	7	35%
Inclusion of wider family	7	35%
Insurance documentation for study	7	35%
PIS Information about risks benefits clearer	7	35%
Wish to have sight of GP letter	7	35%
Suitability of the applicant and supporting staff	7	35%
Evidence supporting use of Baby Bed Box	7	35%
Recruitment of midwives	7	35%
Recruitment location	7	35%
Carrying box with child in it	6	30%
Consent form and correct format titles and signature spaces	6	30%

Information sheet for Baby Bed box	6	30%
Personal data requested by study	6	30%
Video sleep App	5	25%
PIS Vouchers initiative	5	25%
Consent and mental capacity issues	5	25%
Returning box after use	5	25%
High risk nature of participant group	5	25%
Level of input expected from participants	5	25%
Current safer sleep advice	5	25%
Different age range of participant and baseline groups	5	25%
PIS Coercive language	4	20%
Health of mother and baby	4	20%
Comments on safe sleep box supplier	4	20%
PIS GP midwives Health Visitor will be informed	4	20%
PIS Emergency or complaint process PALS	4	20%
PIS Amount of time used in filling out diary and interview for participant	4	20%
Recruiting staff	4	20%
Number of midwives sufficient to manage participants	4	20%
Comments on the trial not being registered	4	20%
PIS Advising participant why they have been selected	3	15%
PIS For midwives	3	15%
PIS Safer Sleep Box information	3	15%
Use of acronyms	3	15%
Consent form and agreement to access medical records	3	15%
Lone working policy	3	15%
Telephone interviews	3	15%
Truthfulness of parents	3	15%
PIS Public involvement	2	10%
Are documents supplied final versions	2	10%
Interview schedule	2	10%
Midwives forms and surveys	2	10%
Consent and general data protection use of study data	2	10%
Consent Form and use of anonymised quotes	2	10%
Consent Form for midwives	2	10%
PIS Addressed to parents rather than mothers	2	10%
PIS Should focus on SIDS	2	10%
PIS Time to consider	2	10%
Consent Form and limits of confidentiality	2	10%
Literacy issues in participant group	2	10%
Potential Cultural Bias in participant group	2	10%
PIS Data protection	1	5%
PIS Implies breastfeeding increases danger to child	1	5%
PIS use of anonymised quotes	1	5%
PIS names reviewing REC	1	5%

Ensure GCP qualified	1	5%
Copies of results for participants	1	5%
Bedding insufficient	1	5%

WHEAT

Opt Out Consent: any mention	12	100%
PIS: Comments on potentially coercive wording	9	75%
Opt Out Consent: Comments on the ethics of the basic principle	8	67%
PIS Suitable description of risks and benefits	7	58%
Opt-Out Consent: how this is to be recorded	7	58%
Opt-Out Consent: Hard copy for patients/clinicians	7	58%
Patient Public Involvement	6	50%
PIS Specific wording	5	42%
PIS Inclusion benefit	5	42%
Data Monitoring Committee	5	42%
Opt Out Consent: appropriate	5	42%
Use of existing case registry by trial	5	42%
Randomisation process	5	42%
Query if this care is standard	5	42%
SSA NHS Sites	4	33%
PIS General Adequacy	4	33%
CONSENT to data usage by parents	4	33%
Additional cannulation	4	33%
24 Hours to consent	4	33%
Why not do retrospective study	4	33%
Study Equipoise	4	33%
PALS details	3	25%
CONSENT Explanation of study	3	25%
Opt Out Consent please Justify	3	25%
No exclusion criteria	3	25%
Inclusion multiple birth infants	3	25%
Study Registration	3	25%
Study has value	3	25%
Study Feed used	3	25%
Statistics supporting study	3	25%
Inclusion withdrawal of feeding or withholding Units	3	25%
Letter headed paper	2	17%
Agreement from site	2	17%
NHS guidance on applying for permission to research	2	17%
Who did peer review	2	17%
Clarification of trial staff GCP	2	17%
PIS Benefits	2	17%

CONSENT list of things consented to	2	17%
Stopping Criteria	2	17%
Further contact bereaved parents	2	17%
Data usage	2	17%
Study results parents informed	2	17%
Recruitment timing	2	17%
Opt-Out Consent NREAP Guidance	2	17%
Opt-Out Consent Other Clinicians agreed	2	17%
Opt-Out Consent	2	17%
Exclusion other Trials	2	17%
Consent by Unit for change in care	2	17%
Study cluster randomisation design	2	17%
Study Funding	2	17%
IRAS form	1	8%
Evidence of Peer Review	1	8%
Staff implementing trial	1	8%
PIS Poster on ward	1	8%
PIS Written for Opt-In Consent	1	8%
PIS With Consent discussion	1	8%
PIS Why has My baby Been Chosen	1	8%
PIS Use NRES Template	1	8%
PIS Transfusion required	1	8%
PIS PPI	1	8%
PIS Name of Reviewing REC	1	8%
PIS Makes Opt Out Consent clear	1	8%
PIS Add study summary	1	8%
CONSENT NRES Guidance	1	8%
Cot labels identifying participants	1	8%
Study Results given before Peer Review	1	8%
Potential distress at feeding withdrawal	1	8%
Burden of participation	1	8%
Withdrawal from Study	1	8%
Recruitment Both Parents	1	8%
Opt Out Consent whole Unit	1	8%
Opt Out Consent burdensome	1	8%
Inclusion of non-English speakers	1	8%
Inclusion 10th Centile infants	1	8%
When Recruitment stopped	1	8%
Longer term study Outcomes	1	8%
Hawthorne Effect	1	8%
